# Effectiveness of a School-Based Interventional Package on Adolescent Obesity in Kanyakumari District

**DOI:** 10.7759/cureus.87076

**Published:** 2025-06-30

**Authors:** Ajitha Rethnam C, Daly Christabel H, K Priscilla, Santhi Letha V, Feby G, Suja T

**Affiliations:** 1 Department of Medical and Surgical Nursing, Sree Mookambika College of Nursing, The Tamil Nadu Dr MGR Medical University, Kulasekharam, IND; 2 Department of Pediatric Nursing, Sree Mookambika College of Nursing, The Tamil Nadu Dr MGR Medical University, Kulasekharam, IND; 3 Department of Medical and Surgical Nursing, C.S.I. Jeyaraj Annapackiam College of Nursing, The Tamil Nadu Dr MGR Medical University, Madurai, IND; 4 Department of Obstetrics and Gynecological Nursing, Sree Mookambika College of Nursing, The Tamil Nadu Dr MGR Medical University, Kulasekharam, IND; 5 Department of Community Health Nursing, St. Xavier’s Catholic College of Nursing, The Tamil Nadu Dr MGR Medical University, Chunkankadai, IND; 6 Department of Community Health Nursing, Sree Mookambika College of Nursing, The Tamil Nadu Dr MGR Medical University, Kulasekharam, IND

**Keywords:** adolescent health, intervention, knowledge questionnaire, obesity, yoga

## Abstract

Background

Overweight and obesity in adolescents have increased significantly in developed and developing countries. The trend, previously exclusive to adults, is now also being seen in young populations. Complementary therapies, such as yoga, are being increasingly recognized as potential interventions for weight control in adolescents. This study aimed to assess the effectiveness of an interventional package using dietary education and yoga in the reduction of obesity among adolescents aged 10-19 years in selected schools in Kanyakumari district, India.

Methodology

A total of 400 overweight or obese students from four private matriculation schools were randomly allocated to an experimental group (n = 200) or a control group (n = 200). The intervention consisted of a guided 1,800-calorie diet education session and 12 weeks of yoga (five days a week). Pre and post-interventional body mass index (BMI), waist-hip ratio (WHR), and well-being scores were measured at three points. Student’s t-test and chi-square test (SPSS version 20.0) were used for statistical analysis, with p-values ≤0.05 being the criterion for significance.

Results

The experimental group showed statistically significant improvements in BMI and WHR at post-intervention measurements compared to controls. Mean BMI decreases were 3.2%, 5.4%, and 7.9% at post-test periods 1, 2, and 3, respectively (Post 1: t = 4.923, p < 0.001; Post 2: t = 6.045, p < 0.001; Post 3: t = 7.837, p < 0.001). Likewise, WHR values reduced consistently. Scores of knowledge and well-being also improved significantly (p < 0.001). Major lifestyle attributes, namely, missing breakfast, red meat consumption, and junk food eating, were strongly related to obesity markers.

Conclusions

The interventional package comprising diet training and yoga was associated with clinically and statistically significant improvements in obesity outcomes in adolescents. These results favor the implementation of such non-pharmacological interventions as part of school health programs to ensure sustainable weight control.

## Introduction

Childhood obesity is considered a serious health problem in the 21st century. Despite being an issue everywhere, it has a much bigger impact on cities in middle- and low-income countries. The increase in the number of overweight children and teens in recent years is very concerning [1]. Overweight and obesity are described by the World Health Organization as having too much body fat, which can harm health [2]. Body mass index (BMI) remains the main way to measure body fat. Being overweight is considered to be a BMI between one and two standard deviations above the mean, while obesity is anything greater than two standard deviations [3]. Most of these conditions develop when the number of calories taken in is not equal to the number of calories burned [4]. Obesity can damage both the body and mind. Children who are in the highest BMI group in early adolescence are five to nine times more likely to be obese adults [5]. In 2021, about 15.1 million (95% uncertainty interval = 13.5-16.8) children and adolescents (aged 5-14), 21.4 million (20.2-22.6) teenagers (aged 15-24), and 172 million (169-174) adults in the United States were considered to be overweight or obese. It is expected that by 2050, 43.1 million children and adolescents and 213 million adults in the United States could be affected [6]. The number of overweight adolescents in India is rising quickly, mainly in cities. According to a 2023 report from the Indian Council of Medical Research, the number of obese urban adolescents has increased by 20% in the past five years due to less physical activity, more time spent on screens, unhealthy eating, and a lack of exercise [7]. Tamil Nadu is one of the five Indian states where childhood and adolescent overweight and obesity are the highest [8]. According to the National Family Health Survey-5, 19.8% of men and 24.4% of women in urban Tamil Nadu were overweight or obese, compared to 8.5% of men and 4.7% of women in rural areas [9].

Preventing and managing obesity in children and adolescents is often achieved through school-based interventions. They provide a planned setting in which students can perform educational, behavioral, and physical activities every day [10]. Yoga has become known as a helpful lifestyle approach for controlling weight, managing stress, and promoting general well-being. Research has shown that the practice, which combines postures, breathing, and meditation, can help adolescents become fitter, manage their emotions, and improve their metabolic health [11]. Some yoga postures are designed to reduce fat in the abdomen and hips, while others help the heart, lungs, and kidneys work better. Yoga helps build muscle strength, improve flexibility, and make people more resilient to stress [12]. Regular exercise has been proven to help people eat better and feel better emotionally, which is why it is useful in obesity prevention [13]. Adding yoga to schools is meant to teach students how to live healthily and support their physical, emotional, and psychological well-being. Studies have found that yoga helps school-age children become stronger, more enduring, and fitter [14]. This study aims to assess the current rate of obesity among adolescents and test whether a yoga-based program can effectively reduce obesity in students in the Kanyakumari district of Tamil Nadu. Community medicine should focus on preventing diseases, and this research encourages the use of early and lasting strategies to address adolescent obesity and support better health in the future.

Aims and objectives

The primary aim of this study is to assess the influence of an interventional package on obesity and excessive weight among students aged 10-19 years in selected schools in Kanyakumari district. To achieve this aim, the study sets out several objectives. First, it seeks to understand the baseline knowledge levels and overall well-being of adolescent students in both the experimental and control groups before the implementation of the interventional package. Second, it intends to calculate the waist-hip ratio (WHR) and BMI of these students before the intervention, providing a clear anthropometric profile across both the intervention and non-intervention groups. Third, the study aims to evaluate the effectiveness of the interventional package in addressing excessive weight and obesity by examining changes in students’ well-being, knowledge, WHR, and BMI after the intervention. Lastly, it seeks to explore the relationship between trends in overweight and obesity and a range of demographic and lifestyle factors, including dietary habits, religion, sex, age, parents’ occupation and education, as well as family type and income. This comprehensive approach is designed to offer valuable insights into the multifaceted nature of adolescent obesity and the potential benefits of school-based interventions. To enhance the long-term public health relevance of the findings, the study will additionally consider the feasibility and sustainability of maintaining positive outcomes over time through school-based reinforcement and follow-up strategies.

Hypothesis

The study is anchored on three primary hypotheses. The first hypothesis posits that the implementation of the interventional package will lead to a statistically significant improvement in the well-being and knowledge levels of adolescent students, with more pronounced improvements anticipated in the experimental group compared to the control group, measured at a 0.05 level of significance. The second hypothesis suggests that there will be a significant reduction in both WHR and BMI among adolescents, particularly within the experimental group, as a result of the intervention, again evaluated at the 0.05 significance level. The third hypothesis proposes a meaningful association between excessive weight and obesity and various demographic factors such as sex, age, religion, and the educational background, occupation, and income level of the students’ parents. Furthermore, it hypothesizes significant correlations between obesity and dietary behaviors, including the frequency of fruit consumption, skipping breakfast, consumption of food from outside the home, water intake, type of cooking oil used, and intake of specific foods such as junk food, bakery items, red meat, butter, cheese, eggs, and milk. These associations are also expected to reach statistical significance at the 0.05 level.

## Materials and methods

A two-group, quasi-experimental study with pre- and post-testing was performed in the chosen schools in Kanyakumari district. The ethics committee at C.S.I. Jeyaraj Annapackiam College of Nursing approved this study, and the Tamil Nadu Dr. MGR Medical University, Chennai, screening committee gave its approval (approval number: 26/6/2014). The research was conducted after seeking necessary permissions from the concerned authorities, such as the principals and the corporate managers of the schools. The chosen schools for this research were L.M.S Matriculation School, Palliyadi; CSI Matriculation School, Nagercoil; Good Shepherd Matriculation School, Marthandam; and Sacred Heart Matriculation School, Padanthalumoodu. All four of these schools are located in urban areas of the Kanyakumari district and offer co-education.

Inclusion criteria

The inclusion criteria for this study encompassed adolescents aged between 10 and 19 years, regardless of gender. Only students who expressed a clear willingness to participate in the research were considered eligible. Additionally, the study specifically targeted adolescent students with a BMI exceeding 25 kg/m², indicative of overweight or obesity. To ensure consistency and relevance to the regional context, only students who were permanent residents of the selected areas within the Kanyakumari district were included in the study population.

Exclusion criteria

The exclusion criteria for this study included students who had been diagnosed with any form of chronic disease, as such conditions could potentially influence weight and health outcomes independently of the intervention. Students with a history of hospitalization or those who had undergone major medical treatments within the past six months were also excluded to avoid confounding health-related variables. Furthermore, any students who demonstrated reluctance or unwillingness to participate in the study were not included, ensuring that only motivated and voluntarily consenting individuals were part of the research sample.

Sample size

A recent research reported that the incidence of health status in selected Tamil Nadu districts was 18% in Tirunelveli, 17.1% in Tiruchirappalli, 23% in Madurai, and 22% in the district of Salem [14]. Therefore, the sample size was determined using the assumption that the average rate of prevalence was 20, where p denotes the prevalence of obesity and overweight = 20%, i.e., p = 0.2. The sample size was calculated using the standard formula: sample size (n) = 4pq/d^2^, where n = sample size, p = prevalence (20), q = 100 − p = 80, and d = 6. Substituting the values into the formula: n = (4 × 20 × 80)/6^2^ = 6,400/36 = 164. The present study included a total of 200 samples to increase the precision of the findings.

Pilot study

A pilot study was conducted among 60 adolescent students who were suffering from weight-related issues, either due to obesity or excessive weight. Of these, 30 adolescent students were assigned equally to both the control and the experimental group. It was affirmed that this research could be implemented, and the instruments were determined to be significant and practically applicable. No confusion, redundancies, or ambiguities existed in any of the questions or tools. Hence, it was inferred that the entire study was possible.

Data collection methods

Purposive sampling was used to choose the samples required for this study. Using the ELIZ Health pathway assessment tool and the standard BMI assessment formula (weight (kg)/height (m²)), the researcher collected baseline data such as height, weight, BMI, and WHR. Out of the 2,582 teenagers who underwent screening, 400 who satisfied the inclusion requirements were chosen from among those whose BMI was higher than 25 kg/m².

A total of 400 teenagers were recruited. Consent was obtained from both the parents and the teenagers by elucidating the study’s objective and nature. A total of 200 participants were equally allocated to the control and experimental groups. The sociodemographic variables encompassed age, gender, parental educational attainment, parental occupational status, religion, family structure, and dietary practices. Clinical parameter Performa comprised four metrics, namely, weight in kg, height in cm, and waist and hip circumference in cm.

The conceptual foundation for this study is grounded in Systems Theory, a modified version of the WHO Global Foundation for addressing adolescent obesity. The interventional measures were executed for the group in three phases. In the first phase, we assembled the chosen pupils at the designated time provided by the principal in an unoccupied classroom. The investigator introduced herself to the participants, established rapport, and clarified the study purpose, which all students understood. Their enquiries were addressed. Subsequently, the researcher administered a pre-test assessing knowledge and well-being using specific questionnaires. The knowledge questionnaire comprised 25 multiple-choice questions designed to assess the participants’ understanding of overweight and obesity, including causes, risk factors, prevention strategies, the importance of physical activity, and healthy dietary habits. Each item had one correct answer, and the total score reflected the level of comprehension, ranging from low to excellent. The well-being questionnaire evaluated emotional, social, and physical aspects of adolescent health using a structured Likert scale format, allowing for a holistic baseline assessment before the intervention.

**Figure 1:**
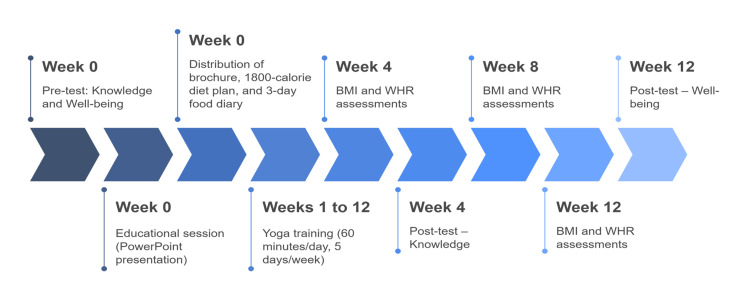
Timeline of intervention phases and assessment schedule. Image created by authors.

Following this, a 60-minute educational session on overweight and obesity was conducted using a PowerPoint presentation in the classroom. Reinforcement education was also provided, accompanied by a discussion and debriefing. Adolescents were provided with a brochure and sample menu of an 1,800-calorie diet, along with a three-day food recording diary for self-evaluation. Both the parents and adolescents were constantly reminded by phone calls to adhere to the dietary modifications. The intervention was implemented in three structured phases, involving educational sessions, supervised yoga training, and periodic assessments. The timeline of the intervention activities, including pre-tests, yoga sessions, and outcome evaluations, is illustrated in Figure 1.

In phase 2, the participants were instructed in yoga, commencing with Surya Namaskar, followed by seven asanas specifically designed for controlling their weight, interspersed with Shavasana, for 60 minutes. The asanas were arranged in a manner that enabled pupils to adhere to them regularly. The asanas’ schedule was as follows: Suryanamaskar (Sun Salutation) involved 14 rounds for 15 minutes; Pavanamuthasana (Supine Asana) was performed for three minutes a single time; Naukasana was practiced for 1.5 minutes; Dhanurasana (Prone Asana) was performed for 1.5 minutes; Vakrasana (Sitting Asana) was held for two minutes on each side, totaling four minutes; Trikonasana (Standing Asana) was performed for two minutes on either side, for an overall duration of four minutes; Parswakonasana was held for two minutes on each side, also totaling four minutes; and Vrikshasana (Balancing Asana) was maintained for a total of 1+1 minutes. Shavasana could be practiced at any time for 30 seconds between the aforementioned poses (Table 1).

**Table 1:** Yoga asanas used in the intervention package.

Asana name	Type of asana	Duration per session	Frequency	Primary target outcome
Suryanamaskar	Dynamic/Full-body	15 minutes (14 rounds)	5 days/week, 12 weeks	Overall conditioning and weight control
Pavanamuktasana	Supine	3 minutes (once)	5 days/week, 12 weeks	Reduces abdominal bloating
Naukasana	Core	1.5 minutes	5 days/week, 12 weeks	Strengthens abdominal muscles
Dhanurasana	Prone	1.5 minutes	5 days/week, 12 weeks	Improves digestion and spinal flexibility
Vakrasana	Seated twist	4 minutes (2 minute/side)	5 days/week, 12 weeks	Aids in fat reduction and digestion
Trikonasana	Standing	4 minutes (2 minute/side)	5 days/week, 12 weeks	Tones waist and improves balance
Parsvakonasana	Standing	4 minutes (2 minute/side)	5 days/week, 12 weeks	Enhances stamina and flexibility
Vrikshasana	Balancing	2 minutes (1 minute/side)	5 days/week, 12 weeks	Improves balance and posture
Shavasana	Relaxation	30 seconds (as needed)	Between poses	Reduces stress and relaxes body

These asanas were practised for 12 weeks, five days a week, with the needed permissions from the school officials. The adolescent students practised these asanas under the proper guidance of the physical education (PE) teacher along with the investigator. Girls and boys were assigned to practice in separate classrooms. In phase 3, a post-test for knowledge was administered after four weeks; post-BMI and WHR were evaluated three times over 12 weeks. Well-being was evaluated after 12 weeks. A knowledge questionnaire was developed to assess understanding of the instructions in the interventional package, especially how to avert and handle overweight and obesity effectively. The instrument comprised 25 multiple-choice questions. These were administered before and after the implementation of the interventional package. The total score attained by the individuals was classified as follows: 0-5 = low comprehension; 6-10 = average comprehension; 11-15 = moderate comprehension; 16-20 = good comprehension; 21-25 = excellent comprehension. The content validity of the questionnaire was established by a panel of five experts in public health and adolescent education. Reliability was confirmed through a pilot test conducted among 30 adolescents, yielding a Cronbach’s alpha of 0.82, indicating good internal consistency.

Statistical analysis

SPSS Statistics version 20 (IBM Corp., Armonk, NY, USA) was used to conduct the statistical analysis. The chi-square test was used to analyze the categorical variables. For continuous variables, Student’s independent t-test was used to compare the two groups, while the paired Student’s t-test was used to evaluate pre- and post-intervention differences within the same group. As the post-tests were administered multiple times, the results were also analyzed using repeated-measures analysis of variance (ANOVA) to confirm consistency over time. The chi-square test was also used to assess associations between pre-test scores and demographic factors. A p-value was considered statistically significant if it was less than or equal to 0.05 (p < 0.05). In addition to statistical significance, effect sizes were calculated to assess the magnitude of changes observed. Cohen’s d was used for t-tests, η² (eta-squared) for ANOVA, and Cramér’s V for chi-square analyses. These metrics were interpreted using standard guidelines (e.g., d = 0.2 small, 0.5 medium, 0.8 large) to understand the practical relevance of results. Where applicable, clinical significance was also considered to contextualize whether statistically significant changes reflected meaningful improvements in adolescent health outcomes.

## Results

The mean age of the control and experimental groups was 14.4 ± 2.1 and 14.1 ± 2.2 years, respectively. A total of 20 (60%) male students and 80 (40%) female students were in the control group, and in the experimental group, there were 88 (44%) male students and 112 (56%) female students. The two groups did not differ significantly from a statistical standpoint (p > 0.05).

Both groups were homogeneous concerning their education, parents’ education and occupation, family type, dietary habits, breakfast skipping, water consumption, oil type used, the rate at which cheese and butter were consumed, egg consumption rate, chicken, milk, red meat, junk food, and bakery items. The heterogeneities were religion and fruit consumption. The groups were comparable and homogeneous (p > 0.05). A pre-tested knowledge questionnaire was utilized to evaluate the understanding of the participants, and a standardized tool for assessing well-being was employed to measure the levels of well-being. The study indicated that in the control group, 145 (72.5%) participants exhibited very poor knowledge, while 55 (27.5%) participants demonstrated poor knowledge in the experimental group. Overall, 148 (74%) exhibited very poor knowledge, while 52 (26%) demonstrated poor knowledge concerning the interventional package.

Among the 200 adolescent students in both groups, 82 (41%) exhibited poor well-being, while 118 (59%) demonstrated moderate well-being. Likewise, in the experimental group, 33 (26.5%) experienced diminished well-being, and 147 (73.5%) exhibited moderate well-being before the implementation of the interventional package. The average knowledge and well-being at the pre-test were not statistically significant (p > 0.05). The post-intervention understanding and well-being of both groups exhibited statistical significance (p < 0.001) (Table 2).

**Table 2:** Knowledge and well-being comparison between both groups pre- and post-test. t = t-value obtained from the independent samples t-test used to assess the statistical significance of the difference between the means of the control and experimental groups. df = degrees of freedom associated with the t-test, calculated based on the sample size. A higher t-value indicates a greater difference between groups, while df helps determine the critical value for statistical significance.​​​​​​​ *** = p < 0.001 (highly significant). No asterisk is added for p-values >0.05, indicating not statistically significant.

Variables	Tests	Controlmean (SD)	Experimentmean (SD)	Difference between means	t	df	P-value
Knowledge	Pre	3.8 (2.0)	4.3 (2.2)	0.5	1.138	258	0.232
	Post	3.8 (2.1)	18.1 (5.1)	14.3	28.155	258	<0.001***
Well-being	Pre	50.8 (13.1)	52.3 (9.6)	1.5	1.267	258	0.112
	Post	51.2 (13.1)	87.3 (10.7)	36.1	24.153	258	<0.001***

In the untreated group of 200 teenagers, 175 (87.5%) were identified as overweight, while 25 (12.5%) were classified as obese. In the experimental group, 116 (89.2%) individuals were classified as overweight, while 14 (10.8%) were categorised as obese. In the control group, 10 (5%) exhibited a normal WHR, 88 (44%) presented an average risk, while 102 (51%) had a WHR classified as a risk according to WHO standards. In the experimental group, 7 (3.5%) exhibited a normal WHR, 69 (34.5%) presented an average risk, while 124 (62%) were categorized as at risk.

**Figure 2:**
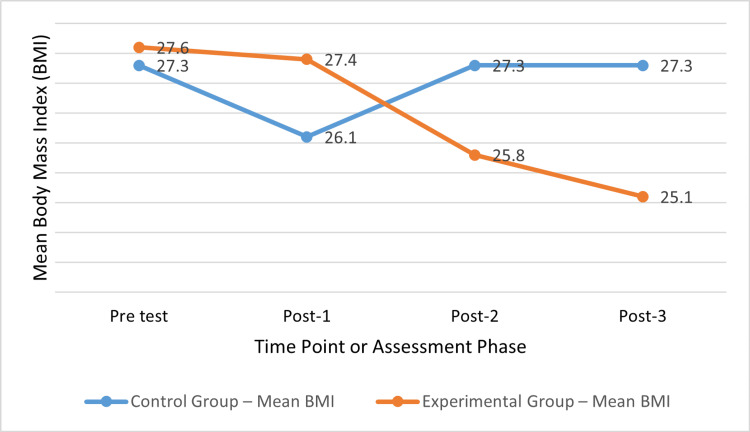
BMI trends across timepoints in control and experimental groups. P-value <0.001. BMI: body mass index

The two groups had mean BMIs of 27.6 ± 1.6 kg/m² and 27.3 ± 1.5 kg/m² at the pre-test. There was no statistically significant difference in the means (p > 0.05). At post-1, the experimental group’s average BMI was 26.1 ± 1.9 kg/m², while the control group’s was 27.4 ± 2.1 kg/m². At the second and third post-tests, the control group’s average BMI was 27.3 ± 2.4 kg/m² and 27.3 ± 2.1 kg/m², respectively. The experimental group’s average BMI was 25.8 ± 2.3 kg/m² at post-2 and 25.1 ± 1.7 kg/m² at post-3. At posts 1, 2, and 3, differences across the groups were statistically significant (p < 0.001) (Figure 2).

**Figure 3:**
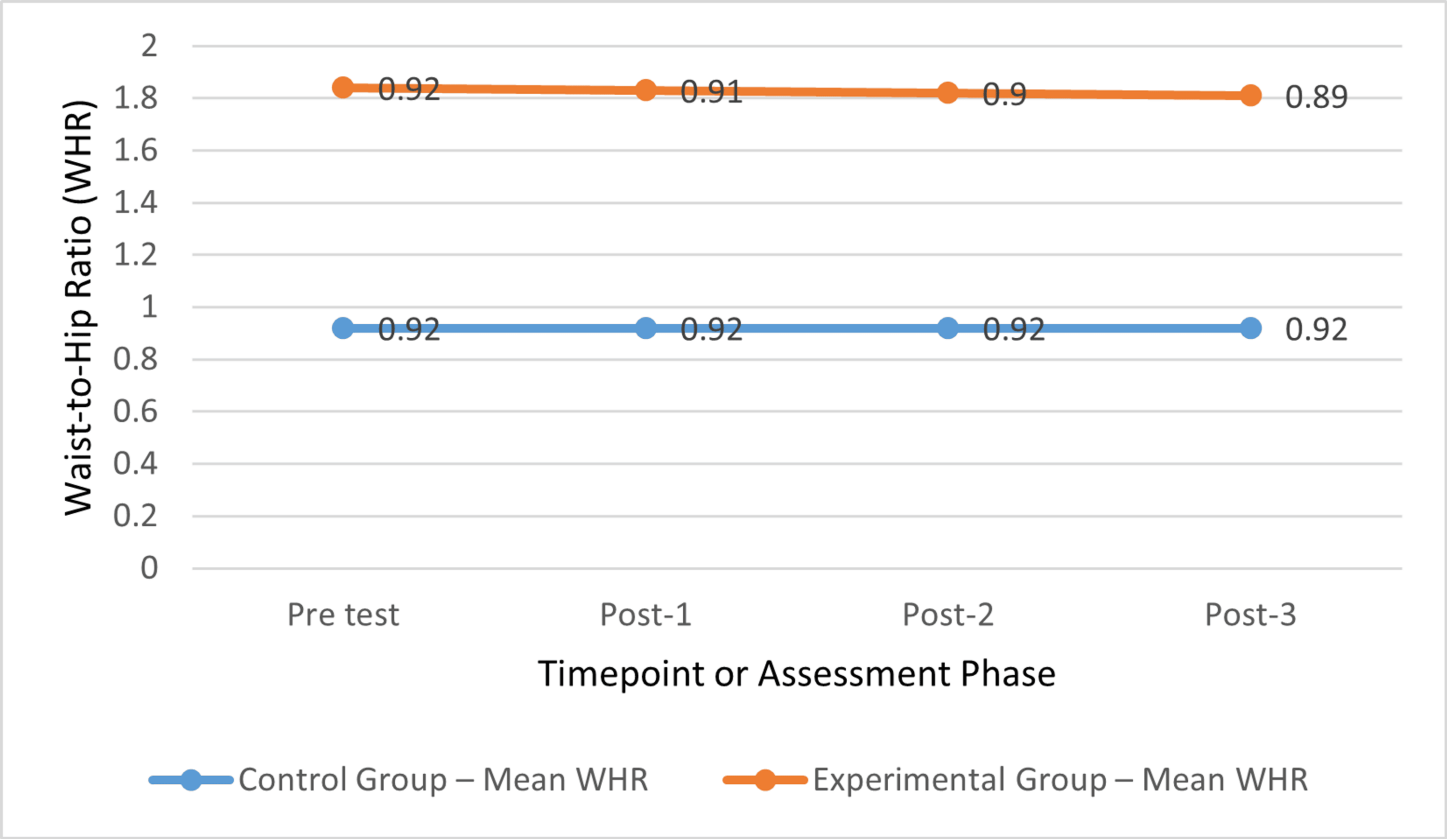
WHR trends over time in control and experimental groups. P-value <0.001. WHR: waist-to-hip ratio

The pre-test mean WHR for both groups was 0.92 ± 0.02 and 0.92 ± 0.01; there was no statistically significant difference (p > 0.05). The mean WHR for the control group was 0.92 ± 0.01 at post-tests 1, 2, and 3. In contrast, the experimental group’s mean WHR at post-tests 1, 2, and 3 was 0.91 ± 0.01, 0.90 ± 0.01, and 0.89 ± 0.01, respectively. The mean differences between the two groups were statistically significant (p < 0.001) (Figure 3).

A notable significance was identified between baseline BMI and WHR in both the experimental and control groups, as well as with variables such as skipping breakfast, consuming bakery items, junk food, red meat, parental education, and reduced fruit intake. Conversely, variables such as religion, gender, age, education levels, and profession did not reveal significant associations.

While the current study demonstrates the short-term efficacy of a combined yoga and dietary education intervention, its long-term sustainability and scalability should also be considered. The integration of such programs into existing school schedules, led by trained PE teachers or health educators, presents a feasible model for wider implementation. Given the low-cost nature of yoga and the adaptability of the educational components, this intervention holds potential for replication in similar sociocultural contexts across India. Future studies could explore follow-up reinforcement strategies to sustain behavior change over time.

## Discussion

Key findings

All demographic characteristics were examined for homogeneity based on the research variables, including attributes such as knowledge levels, overall well-being, BMI, and WHR, to identify significant differences. No significant differences were exhibited by all variables. Hence, it could be affirmed that both groups were identical. From this research, it could be affirmed that the majority of participants in the two groups involved had inadequate information concerning the interventional package. Among the 400 teenagers in both the control and experimental groups, the majority exhibited poor well-being before the introduction of the interventional package. The average knowledge and well-being during the pre-test were not statistically significant (p > 0.05). The post-intervention cognizance and well-being of the control as well as the experimental groups exhibited statistical significance (p < 0.001). The performance in well-being was superior to that in the knowledge test. The anticipated equality was not observed. The disparity between the two test scores indicates that individuals with obesity exhibit a greater interest in themes pertinent to their condition rather than in broader, general topics.

Comparisons with previous research

The study findings were analogous to those of Rawal et al. [15]. This indicated that the proportion of students possessing accurate knowledge regarding good dietary habits and physical activity in their health varied between 27.8% and 76.3%. Participants classified as underweight exhibited a higher prevalence of misconceptions regarding dietary habits and exercise compared to their normal weight counterparts, as evidenced by their inferior understanding of the recommended physical activity levels (37.8% vs. 47.3%, p = 0.003), and a diminished belief that underweight or overweight students experience increased health issues (62.4% vs. 76.3%, p = 0.004). Students with excess weight, conversely, had a greater prevalence of accurate knowledge of the potential correlation between watching television while eating and the risk of excessive weight (46.4% vs. 38.3%, p = 0.002). A calibrated stadiometer and weighing scale were used by the researcher. BMI was determined using a standardized calculator, while a measuring tape was employed to assess waist and hip circumferences. At the pre-test, the average BMI for both groups was recorded as 27.6 ± 1.6 and 27.3 ± 1.5 kg/m². The pre-test average WHR in both groups was recorded at 0.92 ± 0.02 and 0.92 ± 0.01, respectively. The means exhibited no statistically significant difference (p > 0.05). A study aimed to determine the efficacy of an interventional model aimed at altering the lifestyle and nutritional status of children aged 6-12 years through the reduction of BMI [16]. The study indicated no major BMI disparity between the two groups at the commencement of the study (p = 0.40). Furthermore, the children’s BMI assigned to both groups in the sixth month exhibited a statistically significant difference after adjusting for the initial BMI (p = 0.024).

Behavioral trends and correlates

We measured WHR and BMI throughout the four-week intervals of the experimental group intervention period against the standard home and school care received by the control group. The experimental group received beneficial treatment effects because of the intervention. The obese adolescent students from matriculation schools in Kanyakumari district showed increased health risks because their body mass increased during this developmental period, compared to older individuals, as demonstrated by declining BMI and WHR mean scores. The pretest revealed that the control group had a statistically significantly lower mean waist circumference than the study group [17]. The study group demonstrated mean waist circumference measurements of 78.44, 77.65, and 76.81 during post-test 1, 2, and 3, respectively, while the control group recorded 74.60, 74.78, and 74.5, respectively (p < 0.001). The study demonstrated that waist circumference mean values decreased progressively within the study group, but the control group maintained stable mean values, thus confirming that nutritional education interventions produced substantial waist circumference reduction effects.

Comparative intervention studies

A study evaluated the therapeutic value of cognitive behavioral therapy and aerobic exercise for treating obese adolescent students [18]. The mean differences combined with standard deviations between aerobic exercises (Group A) and cognitive behavioral therapy (Group B) demonstrated significant BMI (1.654 ± 0.2186 and 0.841 ± 0.3449) and WHR (0.0282 ± 0.00863 and 0.0131 ± 0.0066) improvements. This study identified a significant link between pre-intervention BMI and WHR in both groups, as well as with variables such as missing breakfast, consumption of junk food, red meat, bakery food items, parental education, and reduced fruit intake. Another study found that junk food intake (p = 0.005) and the purchase of food from street vendors (p = 0.0025) were substantially correlated in adolescents aged 15-19 years [19]. Male participants exhibited a higher frequency of milk and milk product consumption compared to female participants (p < 0.0001). Deficient dietary practices, such as meal omission, consumption of junk food, or purchasing food from street vendors, were correlated with a higher prevalence of participants classified as obese or overweight (p < 0.0001). A higher risk of obesity was linked to factors such as missing breakfast (p < 0.001), eating fewer vegetables (p < 0.001), consuming carbonated drinks more than thrice weekly (p < 0.001), and processed food consumption more than three times a week (p < 0.001) [20].

Limitations

One limitation of the study is that the potency of the interventional package was measured solely using WHR, BMI, well-being, and knowledge. Another limitation is that parental education was restricted to the use of a booklet and reinforcement through phone calls.

## Conclusions

The study findings showed that the adolescent students who received the interventional package stood out in some important ways. The results were much better for overweight adolescents than for obese adolescents. The average score from the interventional package showed a clear improvement in both knowledge and well-being. Similarly, BMI and WHR outcomes supported the effectiveness of the intervention. The results suggest that strategies that follow WHO recommendations, including those that increase knowledge about healthy living and encourage physical activities such as yoga, are important for improving adolescent health and well-being. The findings from this research can be applied to prevent adolescent obesity. Although the intervention achieved its goals in the study, its lasting impact and ability to be applied elsewhere need to be further investigated. More research should be done to check if the health benefits last and to see how the model can be applied in different educational or cultural environments. It is mainly up to school and community health nurses to conduct and monitor these interventions. Health educators, lifestyle coaches, and psychosocial support staff are essential for the intervention’s success, as the study demonstrates. Nonetheless, this study has limitations. It measured the results using only four indicators, i.e., knowledge, well-being, BMI, and WHR, during a short intervention period. Parents were involved mainly by handing out the booklets and calling the students every few weeks. Because of these factors, the intervention’s results should be considered with caution.
